# Neuropsychological changes in *FMR1* premutation carriers and onset of fragile X-associated tremor/ataxia syndrome

**DOI:** 10.1186/s11689-022-09436-y

**Published:** 2022-03-23

**Authors:** Jessica Famula, Emilio Ferrer, Randi J. Hagerman, Flora Tassone, Andrea Schneider, Susan M. Rivera, David Hessl

**Affiliations:** 1 MIND Institute, University of California Davis Health, 2825 50th Street, Sacramento, CA 95817 USA; 2 Department of Psychiatry and Behavioral Sciences, University of California Davis School of Medicine, Sacramento, CA USA; 3 Department of Psychology, University of California Davis, Davis, CA USA; 4 Department of Pediatrics, University of California Davis School of Medicine, Sacramento, CA USA; 5 Department of Biochemistry and Molecular Medicine, University of California Davis School of Medicine, Davis, CA USA; 6 Center for Mind and Brain, University of California Davis, Davis, CA USA

**Keywords:** CANTAB, Fragile X premutation, Tremor, Ataxia, Executive function, FXTAS

## Abstract

**Background:**

Carriers of the *FMR1* premutation are at increased risk of developing a late-onset progressive neurodegenerative disease, fragile X-associated tremor/ataxia syndrome (FXTAS), characterized by intention tremor, gait ataxia, and cognitive decline. Cross-sectional studies to date have provided evidence that neuropsychological changes, such as executive function alterations, or subtle motor changes, may precede the onset of formal FXTAS, perhaps characterizing a prodromal state. However, the lack of longitudinal data has prevented the field from forming a clear picture of progression over time within individuals, and we lack consensus regarding early markers of risk and measures that may be used to track response to intervention.

**Methods:**

This was a longitudinal study of 64 male *FMR1* premutation carriers (Pm) without FXTAS at study entry and 30 normal controls (Nc), aged 40 to 80 years (Pm *M* = 60.0 years; Nc *M* = 57.4 years). Fifty of the Pm and 22 of the Nc were re-assessed after an average of 2.33 years, and 37 Pm and 20 Nc were re-assessed a third time after an average of another 2.15 years. Eighteen of 64 carriers (28%) converted to FXTAS during the study to date. Neuropsychological assessments at each time point, including components of the Cambridge Neuropsychological Test Automated Battery (CANTAB), tapped domains of episodic and working memory, inhibitory control, visual attention, planning, executive control of movement, and manual speed and dexterity. Age-based mixed models were used to examine group differences in change over time on the outcomes in the full sample, and differences were further evaluated in 15 trios (*n* = 45; 15 Pm “converters,” 15 Pm “nonconverters,” 15 Nc) that were one-one matched on age, education, and socioeconomic status.

**Results:**

Compared to Nc, Pm showed significantly greater rates of change over time in visual working memory, motor dexterity, inhibitory control, and manual movement speed. After multiple comparison correction, significant effects remained for motor dexterity. Worsening inhibitory control and slower manual movements were related to progression in FXTAS stage, but these effects became statistically non-significant after correcting for multiple comparisons. Higher *FMR1* mRNA correlated with worsening manual reaction time but did not survive multiple comparisons and no other molecular measures correlated with neuropsychological changes. Finally, trio comparisons revealed greater rate of decline in planning and manual movement speed in Pm converters compared to Pm nonconverters.

**Conclusions:**

Accelerated decline in executive function and subtle motor changes, likely mediated by frontocerebellar circuits, may precede, and then track with the emergence of formal FXTAS symptoms. Further research to develop and harmonize clinical assessment of *FMR1* carriers across centers is needed to prepare for future prophylactic and treatment trials for this disorder.

## Background

Carriers of the fragile X premutation, an allelic variant of the *FMR1* gene with 55–200 cytosine-guanine-guanine (CGG) repeats, are at risk of developing a late-onset, progressive neurodegenerative disease—fragile X-associated tremor/ataxia syndrome (FXTAS). FXTAS is primarily characterized by intention tremor, gait ataxia, and radiological signs of white matter changes in the middle cerebellar peduncles and other regions, as well as brain atrophy. However, the disease frequently presents with comorbid cognitive changes including memory deficits and executive dysfunction and dementia in later stages [1, 2]. A primary underlying molecular mechanism is mRNA toxicity, which leads to protein sequestration, DNA damage and repeat-associated non-AUG (RAN) translation, and ultimately to FXTAS degeneration [3]. By the eighth decade of life, roughly 47% of male premutation carriers develop FXTAS, reaching 75% in those above 80 years [4]; however, clinical presentation is highly heterogeneous in terms of disease severity, age of onset, and course [5–7]. As the gene is X-linked, FXTAS has generally been found to have a lower penetrance and milder clinical presentation in female carriers as compared to males [8, 9].

Though recent studies have pointed toward potential biomarkers that can distinguish which carriers are at greatest risk of developing the FXTAS [10–13], clinical measures to assess individual risk, or even detect the disease in its earliest stages, are limited. Even after disease onset, some patients experience a rapid acceleration in symptom severity, while in others the disease appears to plateau or advance slowly [14, 15]. We still lack reliable methods for predicting which of these scenarios each patient will face.

The neuropsychological profile of premutation carriers of various ages with and without FXTAS has been studied broadly since the disease was first described, with all studies to date taking a cross-sectional approach [16–18]. Among carriers without symptoms of FXTAS, studies indicate that subtle cognitive effects may be present before motor signs develop, and the effects are associated with the extent of white matter changes in the brain [19]. Subsequent well-powered brain volumetric studies show that morphological changes may occur in carriers decades before any formal signs of FXTAS are present [20]. Significantly, functional magnetic resonance imaging (fMRI) studies of premutation carriers have revealed irregular brain activity during working memory [21, 22], associative memory recall [18], and magnitude estimation [22] tasks—further illustrating the neurological underpinnings of these neuropsychological findings. However, the cross-sectional nature of prior work, while extensive, precludes a clear delineation of the prodrome of FXTAS and how the disease first emerges from a neuropsychological perspective. Longitudinal research plays a critical role in identifying which carriers are at the greatest risk of developing the disease, which factors may influence the age of onset and rate of progression of the disease, and which treatment domains may be the most important to target in future trials. Here, we present preliminary results from a longitudinal neuropsychological and neurological study of male fragile X premutation carriers at risk for FXTAS.

## Methods

### Participants

The full study sample consisted of 94 males, 64 with the *FMR1* premutation (Pm), and 30 non-carrier controls (Nc). *FMR1* allele size was confirmed in all participants; premutation carriers had > 54 and < 200 CGG repeats, and controls had < 45 CGG repeats (Table 1). All participants were between 40 and 80 years of age at their first visit (time 1), and the groups did not significantly differ by age, IQ, education, income level, or ethnicity/race (Table 1). Of the 94 participants, 72 had follow-up data at time 2 (50 Pm; interval length = 2.33 ± 0.88 years), and 57 participants had Time 3 data (37 Pm; interval length = 2.15 ± 0.66 years). Note that the reduced sample sizes with subsequent visits do not indicate drop-out as the study is currently ongoing, and future visits at 2-year increments (or longer, given COVID pandemic constraints) will be completed and reported elsewhere.

**Table 1:** Group descriptive statistics

	Control *m* (SD)	Premutation *m* (SD)	*P*
Age in years	57.4 (9.34)	60.0 (8.85)	.198
Education level^+^	6.00 (1.34)	6.13 (1.01)	.612
Income level*	4.45 (1.18)	4.63 (1.80)	.679
FSIQ	122.6 (13.4)	122.8 (14.3)	.933
CGG repeats	29.6 (3.95)	86.08 (19.3)	< 0.001
Psychoactive medication use (%)	20.0	34.4	.159
Race: non-Hispanic Caucasian (%)	80.0	91.0	.087

^+^Education level of 6.0 is equal to a BA/BS degree

*Income levels between 4 to 5 are equal to $75,000 to $150,000 per year household income

*FSIQ* full scale IQ

*FMR1* premutation carriers were recruited primarily through announcements shared by the National Fragile X Foundation, and by referrals from colleagues at other institutions studying children with FXS and their families for research or in clinic. Non-carrier controls were recruited from the Institute’s research registry system, social media announcements, and presentations given to local organizations. Carriers traveled to UC Davis in Sacramento, CA, from various locations in North America, while the non-carrier control population was recruited locally throughout Northern California. During screening, no participants reported having symptoms of tremor or ataxia interfering with activities of daily living (ADLs), nor did they have a history of a significant medical condition or a brain injury. Upon neurological exam, 18 of the carriers (28.1%) showed no clinical signs of FXTAS at study entry (FXTAS stage 0 or 1) but developed clear FXTAS symptoms by visit 2 or 3 (FXTAS stage ≥ 2) and were classified as “Converters.”

All participants provided a full list of their current prescription and over-the-counter medications at each visit. Medication lists were reviewed for those known to have antidepressant, anxiolytic, anticonvulsant, antipsychotic, stimulant, or other known neurologic effects. Groups did not differ significantly on psychoactive medication use (Table 1).

## Measures and procedures

### FXTAS assessment

Diagnosis of FXTAS and the evaluation of disease stage was carried out by a physician using established guidelines [4, 16, 23]. The examination was composed of standardized movement disorder assessments from the Unified Parkinson’s Disease Rating Scale (UPDRS; [24]), International Cooperative Ataxia Rating Scale (ICARS; [25]), Clinical Rating Scale for Tremor (CRST), and the Scale for the Assessment and Rating of Ataxia (SARA; [26]) and review of brain MRI to assess white matter disease associated with FXTAS.

### Neuropsychological assessment

The neuropsychological assessment battery consisted of an intelligence assessment [27, 28], subtests of the Wechsler Memory Scales, Third [29] or Fourth Edition [30], Behavioral Dyscontrol Scale-2 [BDS-2 [31];], Purdue Pegboard Test (Lafayette Instrument, Lafayette, IN), and the Cambridge Neuropsychological Test Automated Battery (CANTAB; Cambridge Cognition, UK). The subtests of the CANTAB included: Paired Associates Learning (PAL), Simple and Five-Choice Reaction Time (RTI), Rapid Visual Processing (RVP), Spatial Working Memory (SWM), Stop Signal Task (SST), and One Touch Stockings of Cambridge (OTS). Together, these tests yielded study measures of spatial memory, episodic memory, auditory working memory, visual working memory, motor dexterity and control, response inhibition, sustained attention, planning, and problem solving, executive control of movement, manual movement speed, and manual reaction time (Table 2).

**Table 2:** Neuropsychological functional domains and variables chosen for each domain

Functional domain	Variable
Episodic memory	
Visual	CANTAB PAL* total errors
Verbal	WMS Logical Memory II - Recall
Working memory	
Visual	CANTAB SWM between errors
Auditory	WMS Letter-Number Sequencing
Inhibitory control	CANTAB SST median correct RT on Go (ms)
Visual attention	CANTAB RVP A' signal detection
Planning	CANTAB OTS problems solved on first choice
Motor function	
Executive control of movement	BDS-2
Manual movement speed	CANTAB RTI 5-choice movement time (ms)
Manual reaction time	CANTAB RTI 5-choice reaction time (ms)
Manual dexterity	Purdue Pegboard (R+L+both hands)

*CANTAB subtest abbreviations are as follows: *PAL* Paired Associates Learning, *SWM* Spatial Working Memory, *SST* Stop Signal Task, *RVP* Rapid Visual Processing, *OTS* One Touch Stockings of Cambridge, *RTI* reaction time

### Molecular measures

Genomic DNA (gDNA) was extracted from 3 mL of peripheral blood leukocytes using standard methods (Qiagen). CGG repeat allele sizing and methylation status by Southern Blot and PCR analysis were measured as previously described [32, 33]. *FMR1* mRNA expression levels were measured by real time qRT-PCR using *FMR1* specific primer and probes as previously reported [34].

### Statistical analyses

We first compared Pm and Nc groups at study entry on measures of age, education level, income, IQ, and use of psychoactive medication to identify any potentially confounding variables for subsequent analyses. Second, we applied age-based mixed models using the MIXED procedure in SAS [35] to compare neuropsychological function of all Pm and Nc and to examine differences between these groups in the rate of change over time for each test. Third, to examine potential effects of changes with FXTAS onset (presence vs absence) and to examine potential effects of molecular variables (*FMR1* CGG size and mRNA) and in combination with increasing age, we again used mixed models, this time including only Pm. Finally, to examine differences between Pm “converters,” “non-converters” and controls, we selected 15 trios (*n* = 45), one-one matched on age, education level, and income and carried out the age-based mixed models described, with a specific focus on differences in the rate of change over time:

$${Y}_{it}={\upbeta}_{0i}+{\upbeta}_{1i}\bullet \mathrm{age}+{\mathrm{c}}_1\bullet {\upbeta}_{0i}+{\mathrm{c}}_2\bullet {\upbeta}_{0i}+{\mathrm{c}}_3\bullet \mathrm{age}\bullet {\upbeta}_{1i}+{\mathrm{c}}_4\bullet \mathrm{age}\bullet {\upbeta}_{1i}+{\mathrm{e}}_{it}$$

where *Y*_*it*_ is the outcome variable for any given individual *i* at time *t*, = β_0*i*_ is the intercept, β_1*i*_ is the linear slope based on age, c_1_ is the contrast testing for differences in the intercept between Pm converters (+ 1) and nonconverters (− 1), c_2_ is the contrast testing for differences in the intercept between Pm converters (+ 1) and Nc (− 1), c_3_ is the contrast testing for differences in the age slope between Pm converters (+ 1) and Pm nonconverters (− 1), c_4_ is c_3_ is the contrast testing for differences in the age slope between Pm converters (+ 1) and controls (− 1), and e_*it*_ is the residual variance. Correction for multiple comparisons was applied using the Benjamini-Hochberg [36] false discovery rate (FDR) method for all effects pertaining to group differences in change over time. Footnotes at the bottom of tables provide guidance for statistical interpretation.

## Results

### Demographic descriptive statistics

Demographic, general intelligence and psychoactive medication use in Pm and Nc at study entry (time 1) are shown in Table 1. The two groups did not differ significantly according to these variables and were generally well above average in intellectual functioning. At study entry, antidepressant medications were reported by 15 Pm and 5 Nc; anxiolytic medications were reported by 9 Pm and 0 Nc; anticonvulsant medications were reported by 5 Pm and 0 Nc; antipsychotic medications were reported by 0 Pm and 0 Nc; stimulant medications were reported by 0 Pm and 1 Nc; and medication with other neurologic effects were reported by 2 Pm and 0 Nc.

### Neuropsychological group differences and comparisons of rates of change with age

Age-based mixed models revealed no significant differences between groups in the value for any variable at age 40 (the youngest age, used as intercept) but showed significantly different rates of change over time between Pm and Nc. Specifically, compared to Nc, Pm showed significantly greater declines in visual working memory, motor dexterity, inhibitory control, and manual movement speed over time (Table 3). However, only the differential rates of change effect in motor dexterity survived multiple comparison correction, while visual working memory and manual movement speed approached significance after correction. Changes in performance over time in the other domains of measurement did not differ between groups.

**Table 3:** Parameter estimates from age-based mixed models (full cohort)

	Estimate (SE)	*t*-value	*P*
Visual episodic memory (PAL errors)			
Intercept	9.47 (2.92)	3.24	.002
Age	.356 (.168)	2.15	.034
Group	− 2.23 (3.94)	− 0.57	.573
Group*age	.224 (.212)	1.06	.294
Verbal episodic memory (WMS LM2 score)			
Intercept	26.89 (2.33)	11.52	.001
Age	.084 (.107)	0.78	.435
Group	− .762 (3.02)	− 0.25	.802
Group*age	− .089 (.137)	− 0.65	.519
Auditory working memory (WMS LNS score)			
Intercept	13.07 (1.05)	12.4	.001
Age	− .059 (.043)	− 1.36	.175
Group	− .515 (1.34)	− 0.38	.702
Group*age	− .018 (.054)	− 0.33	.740
Visual working memory (SWM errors)			
Intercept^1^	31.15 (5.30)	5.88	.001
Age	− .254 (.260)	− 0.98	.330
Group	0^=^ (=)	=	=
Group*age	.438 (.176)	2.49	.014^+^
Motor dexterity (Purdue Pegboard score)			
Intercept^1^	41.17 (1.18)	34.7	.001
Age	− .150 (.061)	− 2.46	.015
Group	0^=^ (=)	=	=
Group*Age	− .200 (.046)	− 4.38	.001*
Inhibitory control^§^ (SST reaction time)			
Intercept	527.8 (31.2)	16.9	.001
Age	1.55 (1.78)	0.87	.358
Group	0^=^ (=)	=	=
Group*age	2.94 (1.40)	2.10	.038
Visual attention (RVP score)			
Intercept	.946 (.018)	52.6	.001
Age	− .001 (.001)	− 0.61	.544
Group	.011 (.023)	0.47	.636
Group*age	− .001 (.001)	− 0.96	.337
Planning (OTS score)			
Intercept	11.43 (.834)	13.7	.001
Age	− .021 (.038)	− 0.55	.582
Group	.147 (1.07)	0.14	.891
Group*age	− .025 (.048)	− 0.51	.608
Executive control movement (BDS-2 score)			
Intercept	24.08 (.693)	34.7	.001
Age	− .041 (.037)	− 1.10	.275
Group	− .054 (.934)	− 0.06	.954
Group*age	− .046 (.048)	− 0.97	.334
Movement time (RT)			
Intercept	217.2 (20.6)	10.5	.001
Age	1.05 (1.15)	0.91	.367
Group	− 15.1 (27.8)	− 0.54	.589
Group*age	3.55 (1.47)	2.41	.018^+^
Reaction time (RT)			
Intercept	297.7 (14.9)	19.9	.001
Age	1.33 (1.03)	1.29	.200
Group	7.77 (19.1)	0.41	.684
Group*age	1.33 (1.28)	1.04	.301

“Intercept” = value at age 40 for controls except for (1), which represents value at age for overall sample. “Age” = change in value per year of age for controls. “Group” = change in intercept value for Pc compared to Nc. “Group*Age” = change in slope value for Pc relative to Nc. “0^=^ (=)” = parameter fixed to zero. *N*_Nc_ = 30, 22, 20 (visit 1–visit 3); *N*_Pm_ = 64, 50, 37 (visit 1–visit 3). * < .05, ^+^ < .10 after Benjamini Hochberg correction for multiple comparisons

### Neuropsychological changes associated with FXTAS stage

The models examining changes associated with FXTAS stage revealed effects for two measures of interest: inhibitory control and manual movement speed. While higher FXTAS stage (≥ 2 on the scale) was associated with poorer inhibitory control scores at age 40, inhibitory control improved slightly over time, with a positive slope reaching statistical significance for those with FXTAS symptoms, potentially due to there being more data from those with symptoms. Manual movement speed was significantly slower at age 40 among those with higher FXTAS stage, but significant change over time was not detected for this measure. In these models, Pm without FXTAS showed significant worsening on several measures including visual episodic memory, auditory working memory, motor dexterity, inhibitory control, and manual movement speed (the “age” factor in Table 4).

**Table 4:** Parameter estimates from age-based mixed models (Pm group by FXTAS status)

	Estimate (SE)	*t*-value	*P*
Visual episodic memory (PAL errors)			
Intercept	7.06 (3.07)	2.30	.025
Age	.572 (.163)	3.51	.001
FXTAS intercept	1.56 (5.38)	0.29	.773
FXTAS slope	− .035 (.239)	− 0.15	.883
Verbal episodic memory (WMS LM2 score)			
Intercept	26.60 (1.86)	14.3	.001
Age	− .004 (.095)	− 0.04	.970
FXTAS intercept	− 4.07 (3.13)	− 1.31	.193
FXTAS slope	.131 (.136)	0.97	.336
Auditory working memory (WMS LNS score)			
Intercept	12.93 (.892)	14.5	.001
Age	− .084 (.041)	− 2.07	.042
FXTAS intercept	− .714 (1.27)	− 0.56	.575
FXTAS slope	.007 (.054)	0.13	.897
Visual working memory (SWM errors)			
Intercept	33.77 (8.15)	4.15	.001
Age	.108 (.375)	0.29	.773
FXTAS intercept	− 17.75 (12.6)	− 1.41	.162
FXTAS slope	.654 (.542)	1.21	.232
Motor dexterity (Purdue Pegboard score)			
Intercept	39.56 (1.62)	24.5	.001
Age	− .245 (.073)	− 3.37	.001
FXTAS intercept	− 2.53 (2.25)	− 1.13	.264
FXTAS slope	.044 (.095)	0.46	.647
Inhibitory control^§^ (SST reaction time)			
Intercept	495.3 (47.2)	10.5	.001
Age	5.44 (2.29)	2.37	.020
FXTAS intercept	186.1 (75.7)	2.46	.016
FXTAS slope	− 6.56 (3.27)	− 2.00	.049
Visual attention (RVP score)			
Intercept	.960 (.016)	61.7	.001
Age	− .001 (.001)	− 1.88	.064
FXTAS intercept	− .010 (.028)	− 0.35	.724
FXTAS slope	.001 (.001)	0.05	.956
Planning (OTS score)			
Intercept	11.03 (.796)	13.8	.001
Age	− .023 (.038)	− 0.61	.544
FXTAS intercept	1.83 (1.14)	1.60	.115
FXTAS slope	− .072 (.049)	− 1.46	.149
Executive control of movement (BDS-2 score)			
Intercept	23.48 (.977)	24.1	.001
Age	− .080 (.041)	− 1.93	.053
FXTAS intercept	− .111 (1.33)	− 0.08	.934
FXTAS slope	.033 (.054)	0.61	.541
Manual movement speed (RT)			
Intercept	185.2 (22.6)	8.18	.001
Age	4.85 (1.17)	4.16	.001
FXTAS intercept	95.0 (38.9)	2.44	.017
FXTAS slope	− 2.97 (1.71)	− 1.74	.086
Manual reaction time (RT)			
Intercept	314.6 (13.0)	24.1	.001
Age	1.49 (.941)	1.58	.118
FXTAS intercept	− 7.95 (28.7)	− 0.28	.782
FXTAS slope	1.86 (1.44)	1.30	.199

“Intercept” = Value at age 40 for individuals without FXTAS. “Age” = change in value per year of age for individuals without FXTAS. “FXTAS intercept” = change in intercept value for individuals with FXTAS. “FXTAS slope” = change in slope value for individuals with FXTAS. “0^=^ (=)” = parameter fixed to zero. Only Pm participants included. *N*_no FXTAS_ = 44, 26, 16 (visit 1 – visit 3); *N*_FXTAS_ = 24, 20, 21 (visit 1 – visit 3). * < .05, ^+^ < .10 after Benjamini Hochberg correction for multiple comparisons

### Neuropsychological changes associated with *FMR1* molecular measures

Models examining the effect of elevated *FMR1* mRNA showed a significant effect on manual reaction time in Pm (higher mRNA, worsening reaction time with age) and a marginally significant negative effect of mRNA on planning. These effects did not survive multiple comparison correction, and CGG repeat number was not significantly associated with any measure of interest.

### Neuropsychological changes in premutation converters, non-converters, and controls (matched trios)

In the domain of visual working memory, Pm converters performed significantly worse than Nc and better than Pm non-converters overall; however, Pm converters had a significantly greater rate of decline than Pm nonconverters (Table 5). Pm converters performed worse than Nc on motor dexterity but their rate of change on this measure did not differ from the other two groups. Pm converters performed better than Pm nonconverters on visual attention and planning overall but showed a significantly greater rate of decline on planning. In contrast, manual movement speed was significantly slower in Pm converters than in Pm nonconverters, and Pm converters became slower over time (*p* = .055). None of these significant effects survived multiple comparison correction (11 test domains, 22 slope comparison *p* values).

**Table 5:** Parameter estimates from age-based mixed models (matched trios)

	Estimate (SE)	*t*-value	Pr > |t|
Visual working memory (SWM errors)			
Intercept	32.37 (5.05)	6.41	<.001
Age	− 0.087 (0.33)	− 0.26	0.796
Conv vs. NConv	− 17.90 (6.92)	− 2.59	0.013
Conv vs. Cont	18.7 (6.98)	2.49	0.011
ConvSlope vs NConvSlope	1.136 (0.46)	2.50	0.017^+^
ConvSlope vs ContSlope	− 0.70 (0.46)	− 1.55	0.128
Motor dexterity (Purdue Pegboard score)			
Intercept	38.97 (1.18)	32.90	<.001
Age	− 0.24 (0.08)	− 3.16	<.001
Conv vs. NConv	1.89 (1.64)	1.15	0. 255
Conv vs. Cont	− 3.37 (1.64)	− 2.06	0.046
ConvSlope vs NConvSlope	− 0.067 (0.11)	− 0.64	0.528
ConvSlope vs ContSlope	− 0.036 (0.10)	− 0.34	0.735
Planning (OTS score)			
Intercept	10.52 (0.56)	18.53	<.001
Age	0.02 (0.04)	0.45	0.654
Conv vs. NConv	2.52 (0.79)	3.21	0.003
Conv vs. Cont	− 1.28 (0.78)	− 1.63	0.110
ConvSlope vs NConvSlope	− 0.15 (0.05)	− 2.94	0.005*
ConvSlope vs ContSlope	0.10 (0.05)	2.01	0.051^+^

“Age” = change in value per year of age, centered at youngest age in this matched sample which is 47.9 years. * < .05, ^+^ < .10 after Benjamini Hochberg correction for multiple comparisons

## Discussion

Here, we present the results of the first longitudinal study of aging *FMR1* premutation carriers, with a focus on neuropsychological functioning. The primary importance of this study’s cohort and design is its emphasis on enrollment prior to the onset of FXTAS and the subsequent tracking of changes in functioning during the emergence of disease. The results demonstrate (a) that age-related declines in executive functions including visual working memory and inhibitory control, as well as manual movement speed, appear to be greater in male premutation carriers than controls without an *FMR1* mutation, and (b) that the onset of the FXTAS disease process (“conversion”) is marked by subtle bradykinesia (slowing of movement) and possibly by changes in frontal-lobe mediated planning ability and working memory. Prior cross-sectional studies have suggested that changes in executive function [21, 37–40] and movement [40] may be key early indicators of imminent or emerging FXTAS; the present longitudinal study documents accelerated *changes* in these parameters over time, providing key support for these initial observations.

Findings of a greater rate of decline in working memory in FXTAS converters are in line with neuroimaging results we have reported in cross-sectional studies of *FMR1* premutation carriers compared to controls. Using functional MRI, we have reported reduced hippocampus activation during recall [18], altered hippocampal-prefrontal function during memory encoding [41], reduced activation in the right ventral inferior frontal cortex and left premotor/dorsal inferior frontal cortex during a verbal working memory task [21], and dysfunctions in the “when” pathway (right temporoparietal junction) during a working memory task requiring temporal order judgments [22]. Using structural neuroimaging, we have also reported significant reductions of fractional anisotropy in multiple white matter tracts, including the middle and superior cerebellar peduncle, cerebral peduncle, and the fornix and stria terminalis—areas that transmit information from the hippocampus and integrate limbic information and monitor valence [42].

Findings in the present study of a significantly greater rate of decline in motor planning, and significantly slower manual movement speed in converters than in the non-converters is also in line with past structural neuroimaging findings. In these cross-sectional studies, we have reported weaker structural connectivity in motor fiber tracts: middle and superior cerebellar peduncles, descending motor tracts (containing the corticospinal, corticobulbar, and corticopontine tracts), and the anterior body of the corpus callosum [43]. In addition, we have reported involvement of subcortical gray structures: thalamus, caudate nucleus, putamen, and globus pallidus (which serve the functions of relaying motor signals to the cerebral cortex, planning and execution of movement, and regulation of voluntary movements) in FXTAS [44]. Finally, in a report of early longitudinal neuroimaging findings from the same cohort described here, we found that decreasing width of the middle cerebellar peduncle (MCP) appeared to be sensitive to early structural changes associated with FXTAS development [11]. The MCP is a structure through which passes the predominant afferent fiber of the corticopontocerebellar pathway—a pathway involved in the communication between the cerebellum and the prefrontal cortex for the coordination and planning of motor responses.

Among our matched trios of controls, Pm converters, and Pm nonconverters, there were two domains in which the converters performed better than nonconverters overall, but the rate of change over time showed a sharper decline in performance among converters compared to nonconverters: visual working memory and planning/problem solving. The trio sample sizes were small, and because we examined multiple domains of function, correction for multiple comparisons made differences hard to confirm. If these findings are upheld with greater numbers and/or replication, they would align with those from a previous study of neuroimaging abnormalities in premutation carriers, in which executive dysfunction and cognitive processing scores were decreased in correlation with white matter changes in the frontocerebellar region of the brain [19]. Interestingly, increased cognitive “load” during standardized walking protocols significantly impairs gait in premutation carriers [45, 46], further highlighting the role of changes in frontocerebellar pathways and functional connectivity in the development of cognitive and motor symptom decline in this population.

An important finding in this study is the motor and executive function changes that occur before and during the onset of FXTAS. These results suggest that prophylactic treatments for molecular abnormalities that have been reported such as oxidative stress and mitochondrial dysfunction [47–49] could be initiated in patients thus identified at high risk, before the onset of FXTAS, to try to stall disease progression. Future targeted treatments for FXTAS and other neurological and neuropsychological symptoms associated with the premutation will need to rely on a well-validated and scalable battery of outcome measures to track treatment response across research and clinical centers. The research presented here points to several neuropsychological domains that may be important to capture in such studies, especially those capturing specific executive functions and cognitive tasks mediated by frontocerebellar activity. Future work should focus on comparing psychometric properties and sensitivity of various outcome measures for premutation carriers and FXTAS in order to refine and then establish a harmonized battery to facilitate collaboration across centers and prepare for future clinical trials.

Among male premutation carriers, it is estimated that approximately 75% will develop FXTAS by the ninth decade of life, with variable ages of onset and severity of course. Thus, the penetrance of the mutation is incomplete and a variety of environmental or secondary genetic factors are likely to affect outcomes. To date, we have limited information about how to predict which carriers will develop FXTAS and when. Limited studies suggest that CGG repeat length is associated with age of symptom onset and age of death among FXTAS carriers [50], but this molecular marker is not an especially powerful predictor. The prodrome of FXTAS, or the early signs or symptoms indicating the imminent onset of the disease, is not yet fully defined. This is an important area of investigation to better identify carriers in need of intervention, prophylactic treatment or lifestyle changes, and monitoring.

A potential weakness of the current investigation is the reliance on touch-screen technology to measure cognition in a population affected by tremor and movement disorder. However, several CANTAB tests (OTS, SWM) are unaffected by reaction time or manual movement accuracy and were sensitive to cognitive changes. Also, the CANTAB has been implemented to detect mild cognitive impairment (MCI) in Parkinson’s disease [51], and it is sensitive to differences between patients with MCI versus those with Alzheimer’s disease [52], demonstrating its broad utility in the assessment of cognitive neurodegeneration. This battery also provides a highly standardized and objectively quantified method that may be scalable for future multi-center studies or clinical trials. We did not include balance or gait measures in this report, whereas ataxia is a primary clinical feature of FXTAS. Detailed gait and balance metrics have been collected and will be reported elsewhere. This study presents the only longitudinal neuropsychological data from premutation carriers in the literature to date. However, because we examined multiple neuropsychological domains in the protocol, requiring multiple comparison adjustments, some group differences in change over time were difficult to confirm. This study focused on male premutation carriers, as fragile X-associated disorders are X chromosome linked, and thus males are more likely to be affected. However, female carriers do develop FXTAS and other neurological symptoms [9, 53], and future longitudinal studies should enroll females to examine phenotypic effects and identify individuals most at risk for neurodegenerative changes, as well as protective factors. Finally, it is important to be aware that individuals identified as “non-converters” might present with signs and symptoms of FXTAS after the period of follow-up in this study, as an average period of 5 years might not be adequate to determine clinical conversion.

## Conclusions

In this prospective longitudinal study, we show that compared to controls, men with the *FMR1* premutation have accelerated decline in manual dexterity and certain domains of executive functioning, including visual working memory, inhibitory control, and that conversion to FXTAS is associated with deterioration in inhibitory control, planning and problem solving, and slowing of manual movement. The findings, in conjunction with prior brain imaging literature, provide critical support for the hypothesis that the prodrome of FXTAS is characterized by executive dysfunction mediated by white matter changes in frontocerebellar pathways. Additional research is needed to select and validate a set of standardized outcome measures that can be used in future multicenter clinical trials of targeted treatments for FXTAS and prophylactic interventions of fragile X carriers who are at increased risk for neurodegeneration.

## Data Availability

The datasets used and/or analyzed during the current study are available from the corresponding author on reasonable request.

## Abbreviations

FXTAS: Fragile X-associated tremor/ataxia syndrome
Pm: Premutation
Nc: Normal control
CANTAB: Cambridge Neuropsychological Test Automated Battery
CGG: Cytosine-guanine-guanine
RAN: Repeat-associated non-AUG
IRB: Institutional Review Board
ADL: Activities of daily living
UPDRS: Unified Parkinson’s Disease Rating Scale
ICARS: International Cooperative Ataxia Rating Scale
CRST: Clinical Rating Scale for Tremor
SARA: Scale for the Assessment and Rating of Ataxia
MRI: Magnetic resonance imaging
BDS-2: Behavioral Dyscontrol Scale, Second Edition
PAL: Paired Associates Learning
RTI: Simple and Five Choice Reaction Time
RVP: Rapid Visual Processing
SWM: Spatial Working Memory
SST: Stop Signal Task
OTS: One Touch Stockings of Cambridge
PCR: Polymerase chain reaction
mRNA: Messenger ribonucleic acid
MCP: Middle cerebellar peduncle
MCI: Mild cognitive impairment
